# Five hypoxia and immunity related genes as potential biomarkers for the prognosis of osteosarcoma

**DOI:** 10.1038/s41598-022-05103-3

**Published:** 2022-01-31

**Authors:** Dachang Liu, Ziwei Hu, Jie Jiang, Junlei Zhang, Chunlong Hu, Jian Huang, Qingjun Wei

**Affiliations:** 1 Department of Orthopedics Trauma and Hand Surgery, Guangxi Medical University First Affiliated Hospital, Guangxi Medical University, Nanning, 530021 China; 2 Department of Spine and Osteopathic Surgery, Guangxi Medical University First Affiliated Hospital, Guangxi Medical University, Nanning, 530021 China; 3 Guangxi Medical University, Nanning, 530021 China

**Keywords:** Predictive medicine, Diagnostic markers

## Abstract

Osteosarcoma accounts for a frequently occurring cancer of the primary skeletal system. In osteosarcoma cells, a hypoxic microenvironment is commonly observed that drives tumor growth, progression, and heterogeneity. Hypoxia and tumor-infiltrating immune cells might be closely related to the prognosis of osteosarcoma. In this study, we aimed to determine the biomarkers and therapeutic targets related to hypoxia and immunity through bioinformatics methods to improve the clinical prognosis of patients. We downloaded the gene expression data of osteosarcoma samples and normal samples in the UCSC Xena database and GTEx database, respectively, and downloaded the validation dataset (GSE21257) in the GEO database. Subsequently, we performed GO enrichment analysis and KEGG pathway enrichment analysis on the data of the extracted osteosarcoma hypoxia-related genes. Through univariate COX regression analysis, lasso regression analysis, multivariate COX regression analysis, etc., we established a predictive model for the prognosis of osteosarcoma. Five genes, including ST3GAL4, TRIM8, STC2, TRPS1, and FAM207A, were found by screening. In particular, we analyzed the immune cell composition of each gene based on the five genes through the CIBERSORT algorithm and verified each gene at the cell and tissue level. Our findings are valuable for the clinical diagnosis and treatment of this disease.

## Introduction

Osteosarcoma is a highly aggressive and malignant common non-hematopoietic primary sarcoma of the bone. It has a poor prognosis and high incidence and is one of the primary causes of cancer-related deaths. Currently, the clinical outcome of patients with osteosarcoma cannot be improved significantly. Therefore, identifying new biomarkers and new therapeutic targets might improve the prognostic outcomes of osteosarcoma^1^.

Hypoxia, a common phenomenon in solid tumors, plays a vital role in the occurrence and development of tumors. Furthermore, it is related to treatment resistance and poor prognosis^2^. Hypoxia increases the risk of invasion, metastasis, treatment failure, and mortality in most solid tumors, including osteosarcoma^3,4^. The rapid growth of tumors causes the spatial disordering of the blood vessel network such that the distance between capillaries exceeds the diffusion range of oxygen, resulting in hypoxia with limited diffusion. Additionally, the microvascular network of the tumor is temporarily absent, and stable blood flow causes fluctuating hypoxia with limited perfusion^5,6^.

Previous studies have implicated hypoxia in the progression of several cancers, such as hepatocellular carcinoma^7^. Hypoxia is also closely related to the occurrence and progression of osteosarcoma, and hypoxia can enhance the metastasis ability of osteosarcoma by affecting the microenvironment of osteosarcoma^8,9^. The hypoxic environment activates multiple transcription factors in tumor cells and induces several downstream signaling molecules to regulate cell proliferation, motility, and apoptosis^10^. Hence, genes related to hypoxia have a prognostic value.

The tumor microenvironment (TME) is a new area of research and contributes to the interaction between tumor cells and immune cells^11^. Extensive research on the TME has demonstrated that tumor-infiltrating immune cells (TIICs) play an important role in the TME^12,13^. The TIICs in the TME can either inhibit or promote the development of tumors. Thus, they play a vital role in tumor dispersion, recurrence, metastasis, and response to immunotherapy^14^. Studies have shown that CD8^+^ T cells, monocytes, and M2 macrophages are involved in the establishment of the immune microenvironment of osteosarcoma^15^. The simultaneous increase in macrophage and B cell infiltration indicates that patients with osteosarcoma have a better prognosis. Type 2 T helper cells and effector memory CD8^+^ T cells affect the metastasis and the chemotherapeutic responsiveness of osteosarcoma cells^16^. Therefore, identifying potential prognostic biomarkers related to tumor-infiltrating immune cells is necessary to improve the existing treatment strategies and select the most appropriate treatment method for patients with osteosarcoma^17^.

In this study, we analyzed the gene expression profile data of osteosarcoma, normal samples, and hypoxia-related genes to obtain the differential genes of hypoxia-related osteosarcoma. Subsequently, we constructed a predictive model for the prognosis of osteosarcoma through univariate COX regression analysis, Lasso regression analysis, and multivariate COX regression analysis and screened five genes related to the prognosis of osteosarcoma, which included ST3GAL4, TRIM8, STC2, TRPS1, and FAM207A. Furthermore, we performed Kaplan–Meier (KM) survival analysis on patients based on the high and low expression of specific genes and the high-risk and low-risk scores, respectively, and analyzed the proportion of 22 TIICs collected from the samples with available osteosarcoma prognostic datasets using the CIBERSORT algorithm.

Additionally, cytokines, chemokines, and growth factors considerably affect the microenvironment of osteosarcoma through the interaction with tumor cells, thereby contributing to the pathogenesis and progression of osteosarcoma^18^. Therefore, we analyzed the relationship between these five genes and the cytokines that were positively related to osteosarcoma. To aid clinical medication, we also studied the drug sensitivity of these five genes. We also evaluated the accuracy of these five genes as new prognostic biomarkers of hypoxia and immune cell infiltration in osteosarcoma at the cellular and histological levels.

## Materials and methods

### Data download

The expression profiles of osteosarcoma specimens and their corresponding survival data were downloaded from the UCSC Xena database (http://xena.ucsc.edu/). Additionally, the gene expression profiles of normal samples were obtained from the GTEx database (https://www.gtexportal.org/home/). Subsequently, log2(x + 1) conversion on all gene expression data was performed. We downloaded the hypoxia-related genes in Gene Set Enrichment Analysis (GSEA, https://www.gsea-msigdb.org/gsea/index.jsp). We used the R (version 4.0.2, R core team, Vienna, Austria, https://www.r-project.org/) program to construct figures and perform all statistical analyses.

### Differentially expressed genes (DEGs) and functional annotation of hypoxia-related genes

To identify the differentially expressed genes between osteosarcoma and normal tissues, we used the Limma package^19^ to identify DEGs in the gene expression matrix using the thresholds of FDR < 0.05 and |logFC|> 1. Additionally, pheatmap, edgeR, and ggplot2 packages were used to construct the volcano and heat maps of the filtered DEGs. Subsequently, based on the GSEA database, we extracted hypoxia-related genes in the expression matrix. We performed differential expression analysis of hypoxia-related genes, and the cut-off value was set as logFC > 0 and FDR < 0.05. Gene Ontology (GO) analysis is a commonly used method for annotating the functions of genes and gene products, including molecular functions, biological pathways, and cellular components^20^. The Kyoto Encyclopedia of Genes and Genomes (KEGG) is a useful resource for gene function system analysis and related high-level genomic function information^21,22^. Next, we used the clusterProfiler package^23^, the org.Hs.eg.db package, the enrichplot package^23^, and ggplot2. The GOplot package^24^ was used to perform GO^25^ and KEGG enrichment analysis^21^ on the DEGs.

### Constructing the prognostic model of osteosarcoma

First, we performed univariate Cox regression analysis to analyze the survival time and survival status of the patient. The selection condition was P < 0.01 obtained from the univariate Cox regression analysis. Next, we screened the eligible genes for the second step of the analysis, i.e., we used the Lasso method to improve the model accuracy and obtain the most important genes to improve its prediction. In the third step, multivariate Cox regression analysis was performed for screening the genes obtained in the second step, based on the threshold of P < 0.05. Finally, the genes and risk scores of the prognostic model of osteosarcoma were obtained.

### ROC analysis

We used the survival package and the survminer package to categorize the patients into two groups (low-risk and high-risk groups).

### Difference analysis and principal component analysis of high-risk and low-risk groups of model genes

First, we used the reshape2 package and the ggpubr package (https://github.com/kassambara/ggpubr) to analyze the differential expression of genes that were used to build the model based on different risk groups (high-risk and low-risk groups). Next, we used the ggplot2 package to analyze the principal components of the different risk groups (high-risk and low-risk groups).

### Prognostic analysis

Patient death was used as the endpoint of prognostic analysis. We used the survival package and the survminer package to divide patients into two groups (high-risk and low-risk groups) according to the different risk values obtained from the model. Next, we conducted survival analysis and plotted the analysis results of the two groups as Kaplan–Meier survival curves. We divided the data into two groups (high-expression and low-expression groups) according to the expression of a single gene. Finally, we conducted a survival analysis of the two groups of patients. The survival package was used to plot the Kaplan–Meier survival curve based on gene expression.

### Risk curve, survival status, and risk heat map

We used the pheatmap package to analyze the risk among all the patients. First, we sorted the results of the analysis according to the level of the model gene risk score. Then, we constructed the risk curve diagram, the risk survival state diagram, and the risk heat map.

### Predicting the survival probability of patients with osteosarcoma

We predicted and tested the risk of the model using the rms package and presented the results in the form of graphs. To evaluate the accuracy of the model, we plotted a calibration graph. We constructed a nomogram to predict the patient’s risk.

### Estimation of the proportion of immune cell types and immune composition of the model genes

First, the CIBERSORT algorithm was used for predicting and quantifying the proportions of immune cells. CIBERSORT is an approach adopted for characterizing cell composition in complicated tissues from its gene expression profile. This can subsequently be applied to estimate immune cell numbers within human osteosarcoma specimens^26^. CIBERSORT implements a machine learning method called Support Vector Regression (SVR), which combines feature selection and powerful mathematical optimization techniques to improve deconvolution performance^27^. Studies have demonstrated that CIBERSORT is more accurate than other methods in solving mixtures of closely related cell subpopulations and unknown cell types (such as solid tissues)^26^. We selected samples with P < 0.05 for the next analysis. The scores of immune cell types of each analyzed sample added up to 1. Finally, we analyzed the immune cell composition of each sample based on the five genes used to construct the model.

### Correlation analysis of osteosarcoma-related cytokines

We performed a correlation analysis to assess the relationship of the five genes, including ST3GAL4, TRIM8, STC2, TRPS1, and FAM207A, with eight cytokines closely related to osteosarcoma, including VEGF, IL-17, IL-6, IL-8, IL-1Ra, TNF-α, IL-34, and TGF-β. We obtained a correlation heat map using the R language and constructed the graphs for the statistically significant correlation analyses (P < 0.05).

### Drug sensitivity analysis

We investigated the relationship between the five genes and drug sensitivity to better guide the clinical use of drugs. We downloaded all the data related to drug sensitivity from the CellMiner database. We used the "impute", "limma", "ggplot2" and "ggpubr" packages of the R programming language to analyze the relationship between the five genes and drug sensitivity.

### Cell culture and treatment

The human osteosarcoma MG63 cell line was provided by Dr. Junlei Zhang of Guangxi Medical University, and the human osteosarcoma SJSA-1 cell line and normal human hFOB1.19 osteoblasts were provided by Otwo Biotech Co. Ltd. (Shenzhen, China). All the cells were cultivated in DMEM F12 medium (Gibco, Shanghai, China) containing 10% fetal bovine serum (FBS; Tianhang, Zhejiang, China) and 1% (v/v) Penicillin/Streptomycin (Solarbio, Beijing, China). The cells were incubated at 37 °C with 5% CO_2_. The medium was replaced every two days.

### Quantitative polymerase chain reaction (qPCR)

The HiPure Total RNA kit (Magen, China) was used for extracting the purified RNA from osteoblasts and osteosarcoma cells for qRT-PCR. Next, cDNA was prepared with 1 μg of the extracted RNA by reverse transcription using the cDNA synthesis kit (Takara, China). The FastStart Universal SYBR Green Master Mix (Roche, Germany) was used to perform qRT-PCR with the LightCycler 480 Sequence Detention System (Roche, Germany) under the following conditions—the initial 10 min at 95 °C, then 15 s at 95 °C for 45 cycles, followed by 60 s at 60 °C. β-actin (Sangon Biotech, China) was used as an internal control, and the 2^−ΔΔCT^ method was used for data analysis. The analysis of each gene was repeated three times. The primer sequences of the target genes are listed in Table 1.

**Table 1:** The primer sequences used in the qRT-PCR experiments.

Gene	Sequence(5′ to3′)
ST3GAL4-F	AGTGATAAGAAGCGGGTGCGAAAG
ST3GAL4-R	TTGGCAGGCTCAGCAGTTTGTC
TRIM8-F	TGGACGCAGAGGTGACAGTGG
TRIM8-R	GGGGTGTGAAGGGGAAGGAGTAG
STC2-F	AGGAGGAAGAGGAGGAGGAGGAAG
STC2-R	CCGCTCGGCACACATGGTTC
TRPS1-F	TCCAGTGATGACCTTCGCAATGTG
TRPS1-R	CCAGGCTTGCTTGGGTGTATGAC
FAM207A-F	GGAAGGACTGGGCGTTCATCAAC
FAM207A-R	ACCTCTCCTGACGGAAGTGACAC
β-actin -F	CCTGGCACCCAGCACAAT
β-actin -R	GGGCCGGACTCGTCATAC

### Western blot analysis

#### Western blot analysis

We performed a detailed protein-level validation of the ST3GAL4, TRIM8, and STC2 genes that differed significantly between osteosarcoma and the normal controls in the PCR results. The β-actin antibody and high sensitive plus ECL luminescence reagent were obtained from Sangon Biotech, China; the ST3GAL4 antibody, TRIM8 antibody, STC2 antibody and Vinculin antibody were obtained from Proteintech. The antibody diluent was purchased from Biyuntian, China. The cells were lysed with RIPA buffer (Beyotime, China) containing protease inhibitor at 4 °C for 30 min. An equal amount of protein samples of hFOB1.19 cells, MG63 cells, and SJSA-1 cells were separated by SDS-PAGE and transferred to the PVDF membrane (Millipore, Germany). The samples were rinsed with the Tbst buffer solution, incubated with the primary antibody for 12 h, washed with the Tbst buffer solution, incubated with the HRP-labeled secondary antibody (Sangong Bioengineering Co., Ltd., China) for 1 h, incubated with the high sensitive plus ECL luminescence reagent for 2 min, and analyzed using the Ultra-sensitive multifunctional imager (Amersham Pharmacia GE, America) to visualize the protein. β- actin was used as an internal control for the TRIM8 gene. Vinculin was chosen as an internal control for the ST3GAL4 and STC2 genes as the protein molecular weights of these genes are similar to those of β-actin.

### Immunohistochemistry

To verify the accuracy of the analysis, we used immunohistochemistry to characterize the selected genes based on osteosarcoma and the adjacent tissues by tissue staining. The ST3GAL4 antibody used for immunohistochemical staining was obtained from Proteintech (https://www.ptgcn.com, catalog number: 13546–1-AP), while the TRIM8 antibody was obtained from Absin (https://www.absin.cn, item number: abs128180). The STC2 and TRPS1 antibodies were obtained from Abcam (https://www.abcam.cn, item number: ab255610; ab209664) and the FAM207A antibody was procured from NOVUS (https://www.novusbio.com, Item No.: NBP1-90,676). Both the osteosarcoma tissue section and the normal tissue section adjacent to the cancer were treated by performing a series of steps such as dewaxing, antigen retrieval by microwave, sealing, primary antibody incubation, and secondary antibody incubation. Finally, the stained osteosarcoma tissue section and the adjacent cancer tissue section were observed under a microscope. Normal tissues were sectioned and their protein expression was determined.

### Ethics approval and consent for participation

The present work was approved by the Ethics Committee from the First Clinical Affiliated Hospital of Guangxi Medical University.

### Consent for publication

The co-authors in this work provided the consent for publication.

## RESULTS

### Data downloading and analysis of differentially expressed genes

**Figure 1:**
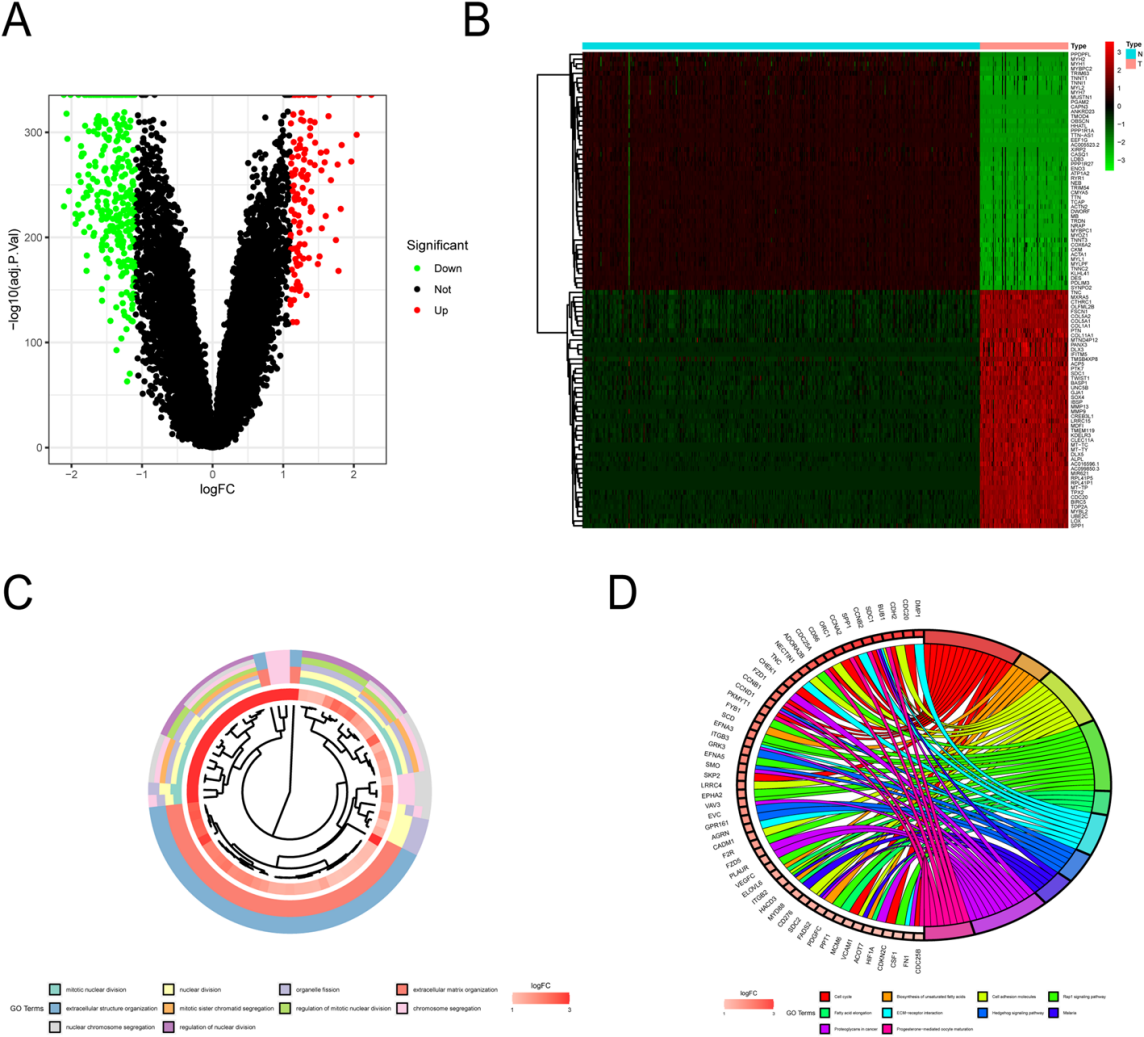
Analysis of differentially expressed genes related to osteosarcoma and enrichment analysis of differentially expressed genes related to hypoxia. (**A**) The volcano plot of DEGs. The red dots indicate genes that have a high expression level, the green dots indicate genes that have a low expression level, and the black dots indicate genes that do not meet our requirements. (**B**) The heat map of DEGs. In the Type column, blue represents normal samples, and red represents tumor samples; red in the graph indicates genes with high levels of expression, while green indicates genes with low levels of expression. (**C**) The Gene Ontology (GO) enrichment analysis of hypoxia-related genes. Different color modules represent different GO terms. The color in the innermost circle represents the logFC value of the gene. (**D**) The top 10 Kyoto Encyclopedia of Genes and Genomes (KEGG) pathways related to hypoxia. The different color modules on the right side of the graph represent different KEGG pathways; the left side represents genes, and the intensity of red indicates the logFC value.

We downloaded the expression profile data of 88 osteosarcomas and their corresponding survival data from the UCSC Xena database (https://xena.ucsc.edu/). The GTEx database (https://www.gtexportal.org/) was used to download the expression profile data of 396 normal samples (normal controls). A total of 2,678 hypoxia-related genes in 41 hypoxia-related gene sets were downloaded from the GSEA database. We performed a differential analysis of 54,751 genes in the gene expression matrix composed of 88 tumors and 396 normal samples and obtained 359 DEGs, which were visualized as heat maps and volcano maps (Fig. 1A,B). Subsequently, we extracted hypoxia-related gene expression profile data and performed differential analysis to obtain 359 upregulated hypoxia-related DEGs.

### GO enrichment analysis and KEGG pathway enrichment analysis

The R programming language was used to analyze hypoxia-related differential genes and obtain the results of the GO enrichment analysis. We showed that the first 10 items (Fig. 1C) were primarily distributed in the mitotic nuclear division, nuclear division, and extracellular matrix organization. The KEGG pathways were mainly enriched in the cell cycle, the Rap1 signaling pathway, and the Hedgehog signaling pathway (Fig. 1D).

### Construction of a prognostic model of osteosarcoma

**Figure 2:**
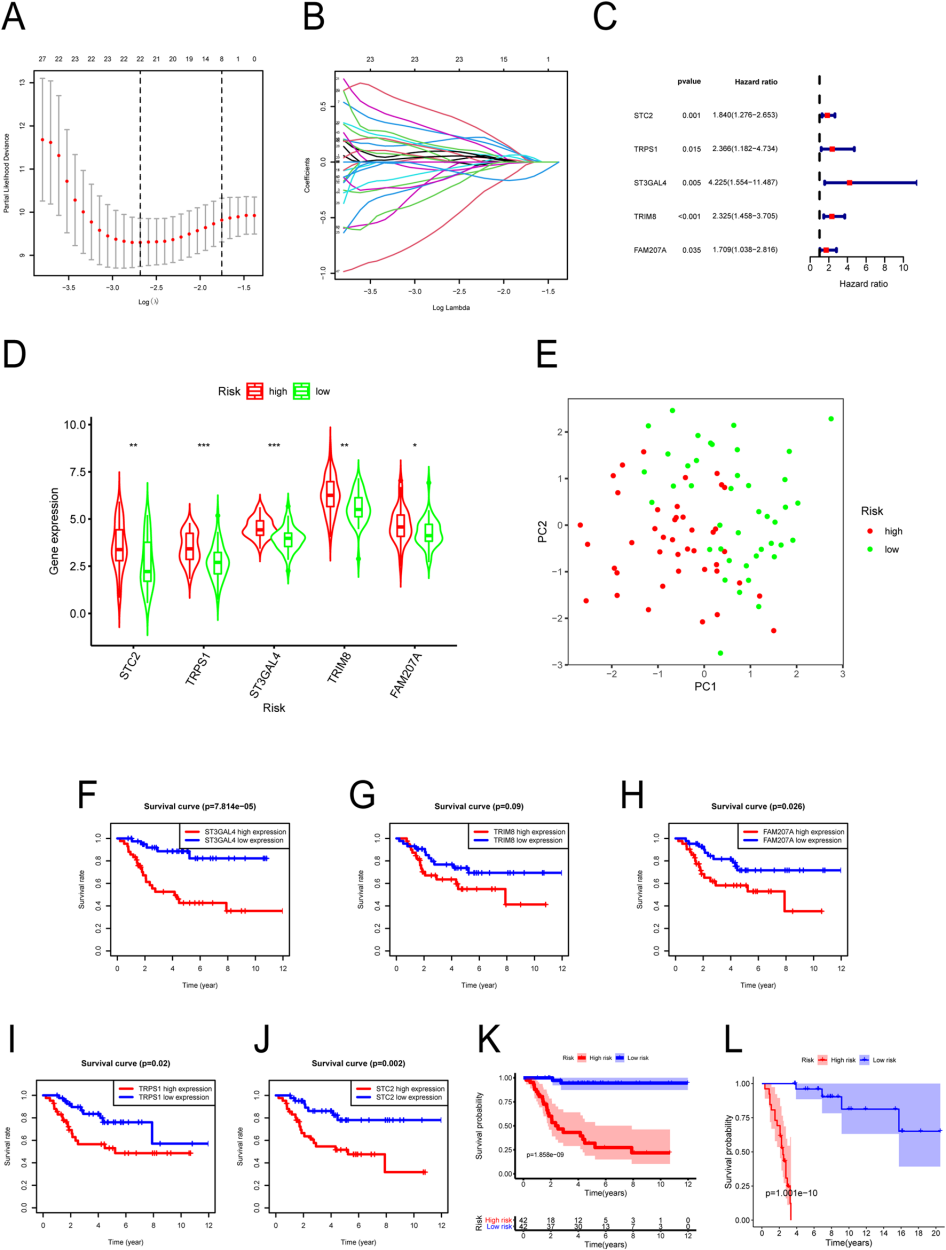
Construction of a prognostic model for osteosarcoma and the survival analysis of the screened genes. (**A**) Ten-fold cross-validation of Lasso regression model adjustment parameter selection (nfold = 10). (**B**) The Lasso coefficient curve of the 22 genes that were included in the analysis. (**C**) The forest plot shows that the multivariate Cox regression analysis of STC2, TRPS1, ST3GAL4, TRIM8, and FAM207A is statistically significant (P < 0.05). (**D**) The violin plot shows the expression difference of the five genes in the high-risk group and the low-risk group. (**E**) The main component analysis chart; the red dots are high-risk patients and the blue dots are low-risk patients. (**F**–**J**) The survival curves were plotted based on the high and low expression of ST3GAL4, TRIM8, FAM207A, TRPS1, and STC2 genes, respectively. (**K**) A survival curve was plotted based on the high and low risks of the genes used to construct the model. (**L**) A survival curve was plotted in the validation dataset based on high and low risk for the genes used to construct the model.

After the preliminary univariate COX regression analysis, the remaining 165 hypoxia-related genes met the requirements of the study. Next, we performed Lasso regression analysis to further improve the accuracy of the model. Among the genes retained in the previous step, 22 genes met the conditions of Lasso regression analysis (Fig. 2A,B). Therefore, we selected those 22 genes for the next analysis. Finally, we performed multivariate Cox regression analysis, and only five genes met our screening conditions, which included ST3GAL4, TRIM8, STC2, TRPS1, and FAM207A (Fig. 2C). We calculated the risk score for each sample and divided all cases into high-risk and low-risk groups according to the mean value of the risk score.

### Difference analysis and principal component analysis (PCA) of the model genes in both groups

We next analyzed the expression of the genes used to construct the model in low-risk and high-risk groups. As shown in the violin plot (Fig. 2D), the expression of ST3GAL4, TRIM8, STC2, TRPS1, and FAM207A genes in the high-risk group increased relative to that in the low-risk group (P < 0.05). The PCA plot (Fig. 2E) showed differences between the two groups. Most cases of the high-risk group were distributed on the right of the PC1 axis, while most cases of the low-risk group were distributed on the left of the PC1 axis; the two groups could be differentiated.

### Subsistence analysis

We analyzed the survival data at different levels. We first plotted the Kaplan–Meier survival curve based on the high and low expression of the five genes used to construct the model. Figure 2F–J shows that the five-year survival rate of osteosarcoma cases with high expression of ST3GAL4, TRIM8, FAM207A, TRPS1, and STC2 was lower than that of low-expressing cases (P < 0.05). We next divided the patients into high-risk and low-risk groups based on the high-risk and low-risk groups of the prognosis model. Figure 2K,L show that the survival rate of osteosarcoma patients in the high-risk group was considerably lower than that of the patients in the low-risk group (P < 0.001).

### ROC diagnostic curve

**Figure 3:**
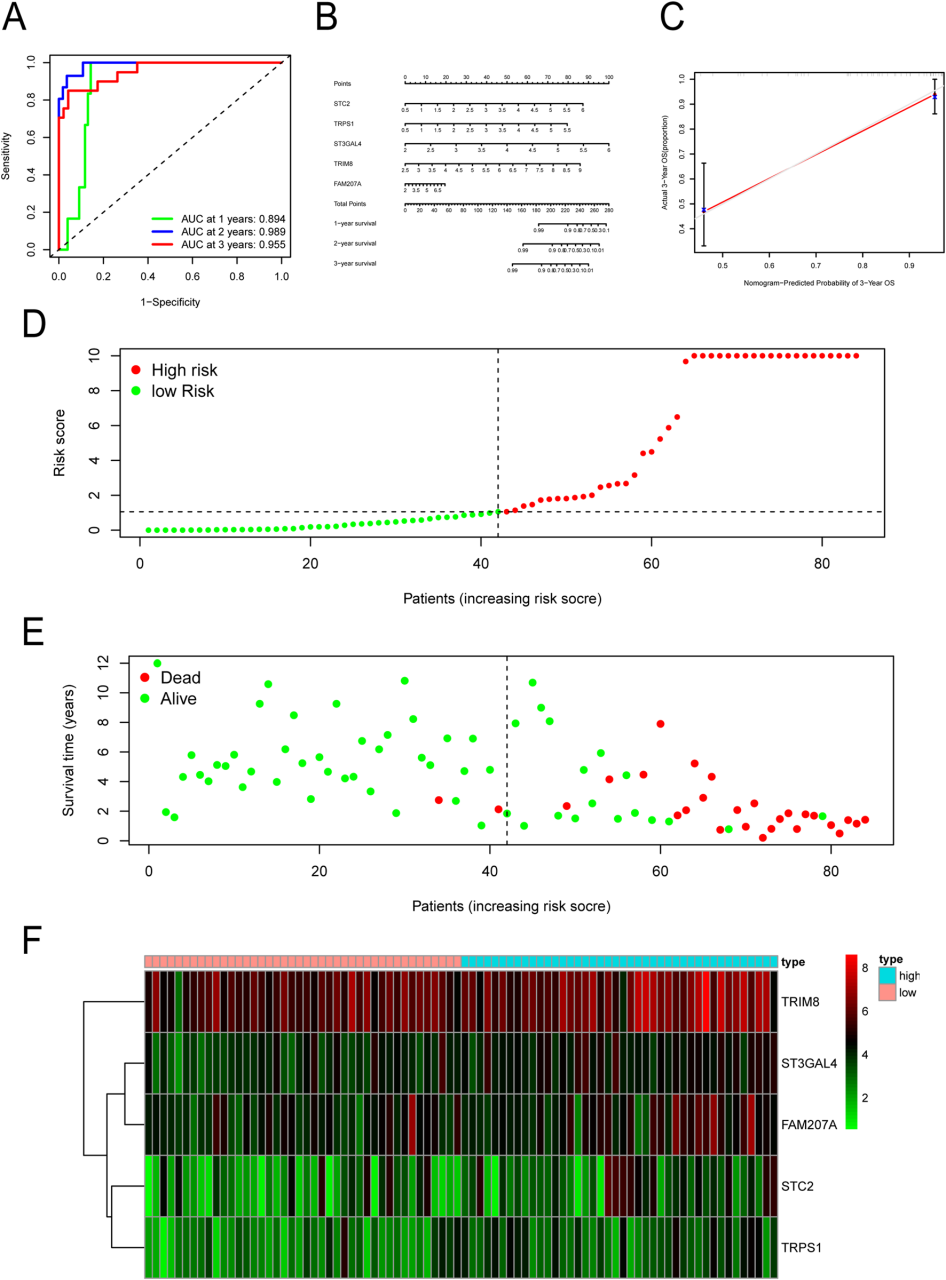
The prediction of the survival probability of the patients with osteosarcoma and the correlation between the expression levels of the five genes in the risk model. (**A**) ROC diagnostic curve. The AUC values used to predict the survival for one, two, and three years were greater than 0.8. (**B**) The nomogram of the five genes that were used to predict the survival for one, two, and three years, based on the model. (**C**) The calibration chart represents the predicted overall survival for three years. There was a good agreement between the actual and predicted survival rates. (**D**) The cases are ranked from low risk to high risk. (**E**) A scatter plot based on the time of death and risk of each case. (**F**) Heat map of risk showing the expression of the five genes from low risk to high risk; they all rise in sequence.

To test the accuracy of the prognostic model, we constructed the ROC diagnostic curve to test the three-year survival probability. The area under the ROC curve (AUC) was 0.894, 0.989, and 0.955 for one, two, and three years, respectively; all AUC values were greater than 0.05 (Fig. 3A). This further confirmed the accuracy of the model we constructed.

### Risk chart display

We used the model to calculate the risk values of all cases and displayed them from low to high (Fig. 3D). The survival time of the patients in the low-risk group was generally longer than that of the patients in the high-risk group (Fig. 3E). The risk heat map (Fig. 3F) showed that the gene expression of ST3GAL4, TRIM8, STC2, TRPS1, and FAM207A increased from low-risk to high-risk groups, indicating that the five genes in this study were related to osteosarcoma.

### Calibration chart and nomogram

To predict the 1-year, 2-year, and 3-year survival rates of patients with osteosarcoma, we used a nomogram (Fig. 3B) to add the values of various indicators in the figure to obtain the final score, which corresponded to the predicted survival rate. Next, to evaluate the accuracy of the constructed prognostic model, we made a calibration chart (Fig. 3C). The calibration chart showed a favorable agreement between the actual and the predicted survival.

### Immune cell composition of each sample and immune cell composition and correlation heat map of the model gene

**Figure 4:**
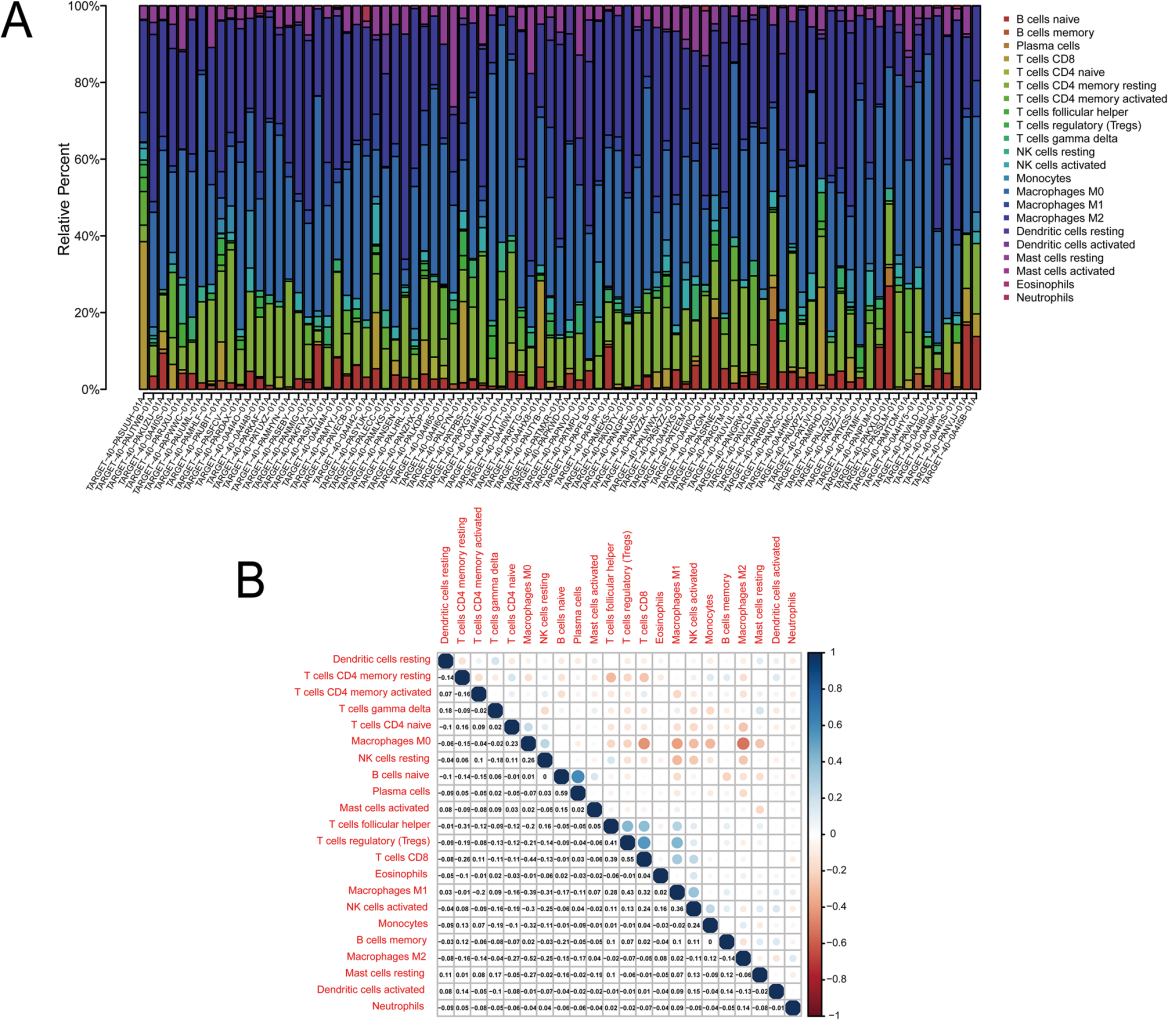
Immune cell composition and correlation analysis of osteosarcoma. (**A**) The proportion of 22 immune cells in osteosarcoma. (**B**) The correlation matrix between various immune cells; red represents positive correlation, and blue represents negative correlation, a darker color indicates stronger correlation.

The results of the analysis using the CIBERSORT software showed that osteosarcoma is related to a variety of immune cells. We constructed an immune cell composition map (Fig. 4A) and a correlation heat map (Fig. 4B) of the samples with statistically significant differences (P < 0.05). Among these, 83 cases in Fig. 4A were statistically significant.

**Figure 5:**
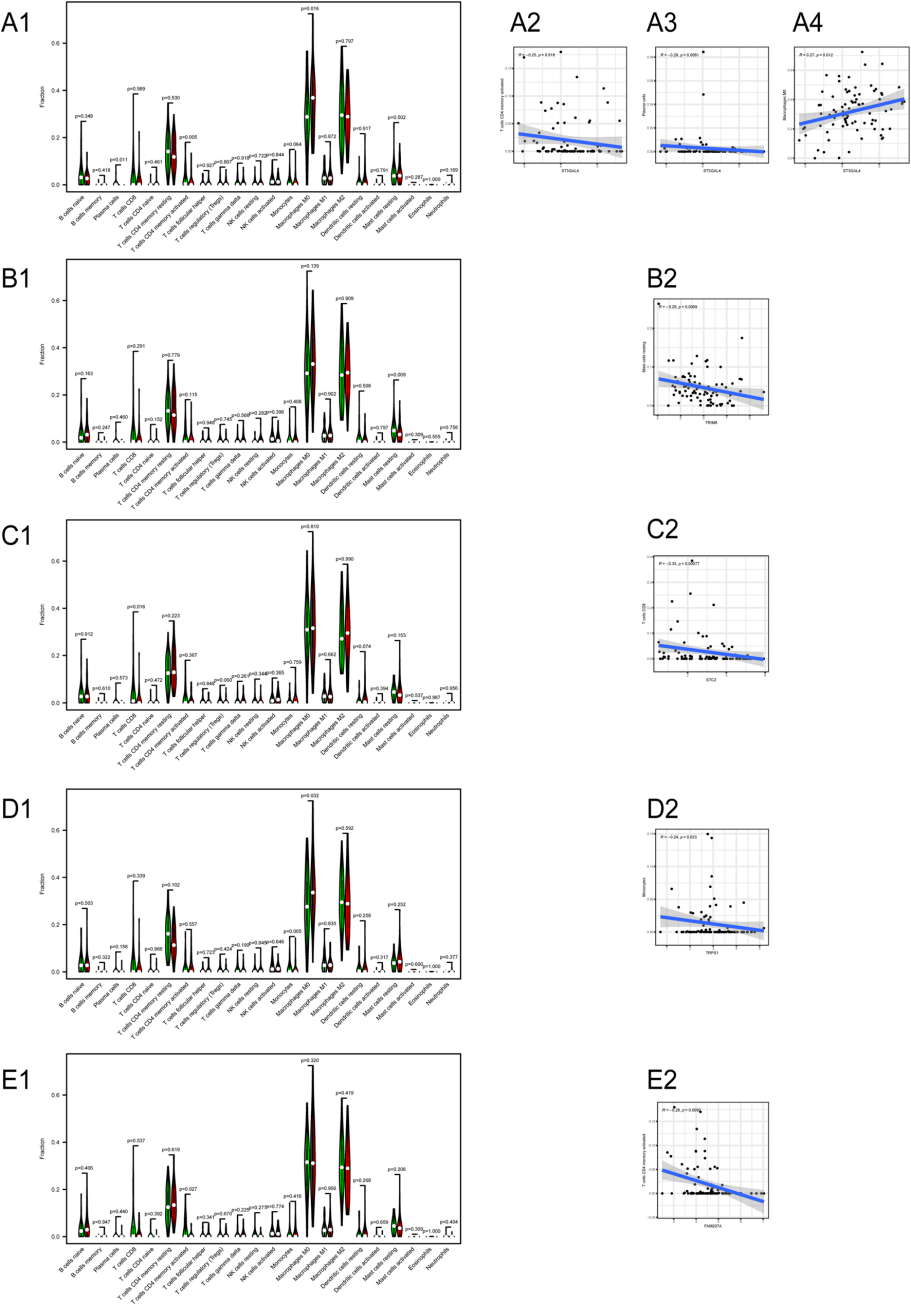
Violin diagram and correlation diagram of immune cells. (**A1**) Violin diagram showing the difference in the degree of infiltration of 22 immune cells based on the ST3GAL4 gene in osteosarcoma; (**A2**–**A4**) indicate the correlation between ST3GAL4 and three different immune cells. (**B1**) The difference in the infiltration of 22 kinds of immune cells based on the TRIM8 gene in osteosarcoma. (**B2**) The infiltration correlation between TRIM8 and resting mast cells. (**C1**) The difference in the infiltration of 22 kinds of immune cells based on the STC2 gene in osteosarcoma. (**C2**) The correlation between STC2 and CD8^+^ T cell infiltration. (**D1**) The difference in the infiltration of 22 kinds of immune cells based on the TRPS1 gene in osteosarcoma. (**D2**) The infiltration correlation between TRPS1 and monocytes. (**E1**) The difference in the infiltration of 22 immune cells based on the FAM207A gene in osteosarcoma. (**E2**) The correlation between STC2 and memory-activated CD4^+^ T cell infiltration.

The violin plot showed that the immune cell composition based on ST3GAL4 (Fig. 5A1) activated the CD4^+^T memory cells, plasma cells, and M0 macrophages (P < 0.05). Among them, ST3GAL4 gene expression was negatively related to the plasma cells and activated mast cells (Fig. 5A2, R =  − 0.25, P = 0.018; Fig. 5A3, R =  − 0.29, P = 0.0061). ST3GAL4 showed a positive relationship with the M0 macrophages (Fig. 5A4, R = 0.27, P = 0.012). Among them, plasma cells and activated CD4^+^T memory cells have important inhibitory effects on tumor growth. The M0 macrophages are closely related to the poor prognosis of osteosarcoma. The violin plot (Fig. 5B1) showed that TRIM8 gene expression was negatively related to resting mast cells (Fig. 5B2, R =  − 0.29, P = 0.0069). Resting mast cells can affect tumor growth by releasing inflammatory mediators^28^. The violin plot (Fig. 5C1) showed that the STC2 gene expression was inversely proportional to CD8^+^ T cells (Fig. 5C2, R =  − 0.35, P = 0.00077), which was statistically significant. The CD8^+^ T cells can kill osteosarcoma cells directly. The violin plot (Fig. 5D1) showed that TRPS1 gene expression was inversely proportional to monocytes (Fig. 5D2, R =  − 0.24, P = 0.023). Monocytes could also mediate tumor cell apoptosis, including osteosarcoma. The violin plot (Fig. 5E1) showed that the FAM207A gene expression was inversely proportional to activated CD4^+^T memory cells (Fig. 5E2, R = –0.28, P = 0.0092). Therefore, these five genes are closely related to the poor prognosis of osteosarcoma.

### Correlation analysis of osteosarcoma-related cytokines

**Figure 6:**
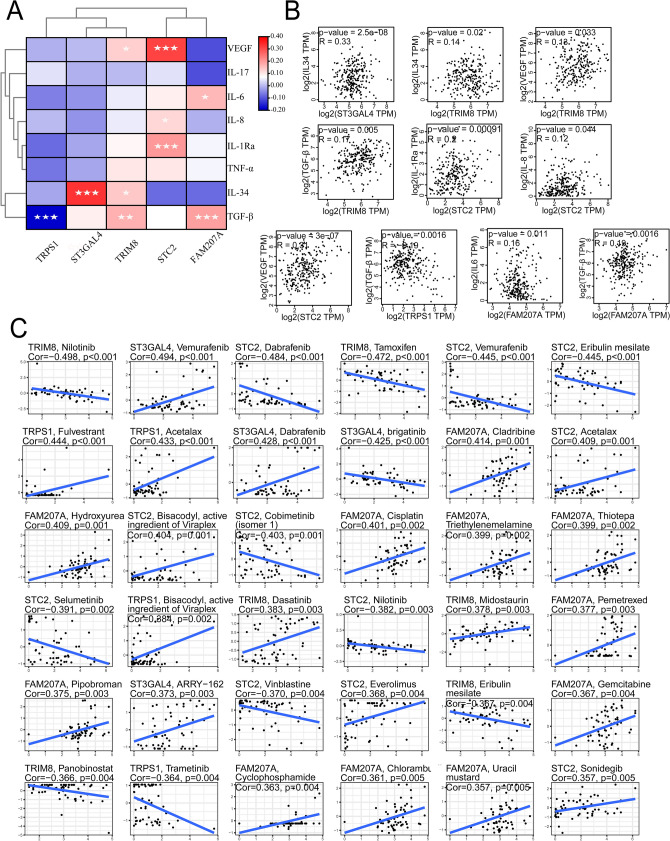
Correlation analysis of osteosarcoma-related cytokines and drug sensitivity analysis of five genes. (**A**) Heat map of the correlations between the five genes and osteosarcoma-associated cytokines, where red represents positive correlations and blue represents negative correlations. (**B**) The statistically significant correlation plots from the correlation analysis of the five genes and osteosarcoma-associated cytokines. (**C**) The drug sensitivity analysis of the five genes. "Cor value > 0" indicates that the gene is positively correlated with the sensitivity of the drug, and "Cor value < 0" indicates that the gene is negatively correlated with the sensitivity of the drug. When the relationship is positive, a higher expression value of the gene indicates stronger sensitivity of the corresponding drug. For a negative correlation, a higher gene expression value indicates weaker sensitivity of the corresponding drug. (" * " represents P < 0.05, " ** " represents P < 0.01, and " *** " represents P < 0.001).

Correlation analysis revealed that ST3GAL4 and IL-34 were positively correlated. TRIM8 was positively correlated with VEGF, IL-34, and TGF-β, whereas STC2 was positively correlated with VEGF, IL-8, and IL-1Ra. FAM207A was positively correlated with IL-6 and TGF-β; TRPS1 was negatively correlated with TGF-β (Fig. 6A,B). The cytokines VEGF, IL-17, IL-6, IL-8, IL-1Ra, TNF-α, and IL-34 can promote the proliferation of osteosarcoma cells, and TGF-β exerts dual effects on tumors; they inhibit tumor growth in the early stage and promote tumor growth in the late stage. Therefore, the five genes including ST3GAL4, TRIM8, STC2, TRPS1, and FAM207A promoted the growth of osteosarcoma by influencing osteosarcoma-related cytokines, which resulted in a worse prognosis.

### Drug sensitivity analysis

After the drug sensitivity analysis of the five genes, we found that they were closely related to the drug sensitivity of various drugs. Among them, ST3GAL4 showed a positive correlation with Vemurafenib, Dabrafenib, and ARRY-162, and a negative correlation with Brigatinib; TRIM8 showed a positive correlation with Dasatinib and Midostaurin, and a negative correlation with Nilotinib, Tamoxifen, Eribulin mesilatedeng, and other drugs; STC2 showed positive correlations with Acetalax, Bisacodyl, and other drugs, and negative correlations with Dabrafenib, Vemurafenib, and other drugs; TRPS1 showed a positive correlation with Fulverstrant, Acetalax, and Bisacodyl and a negative correlation with Trametinib; FAM207A showed a positive correlation with the drug sensitivity of Cladribine, Hydroxyurea, and other drugs (Fig. 6C).

### Quantitative polymerase chain reaction (qPCR)

**Figure 7:**
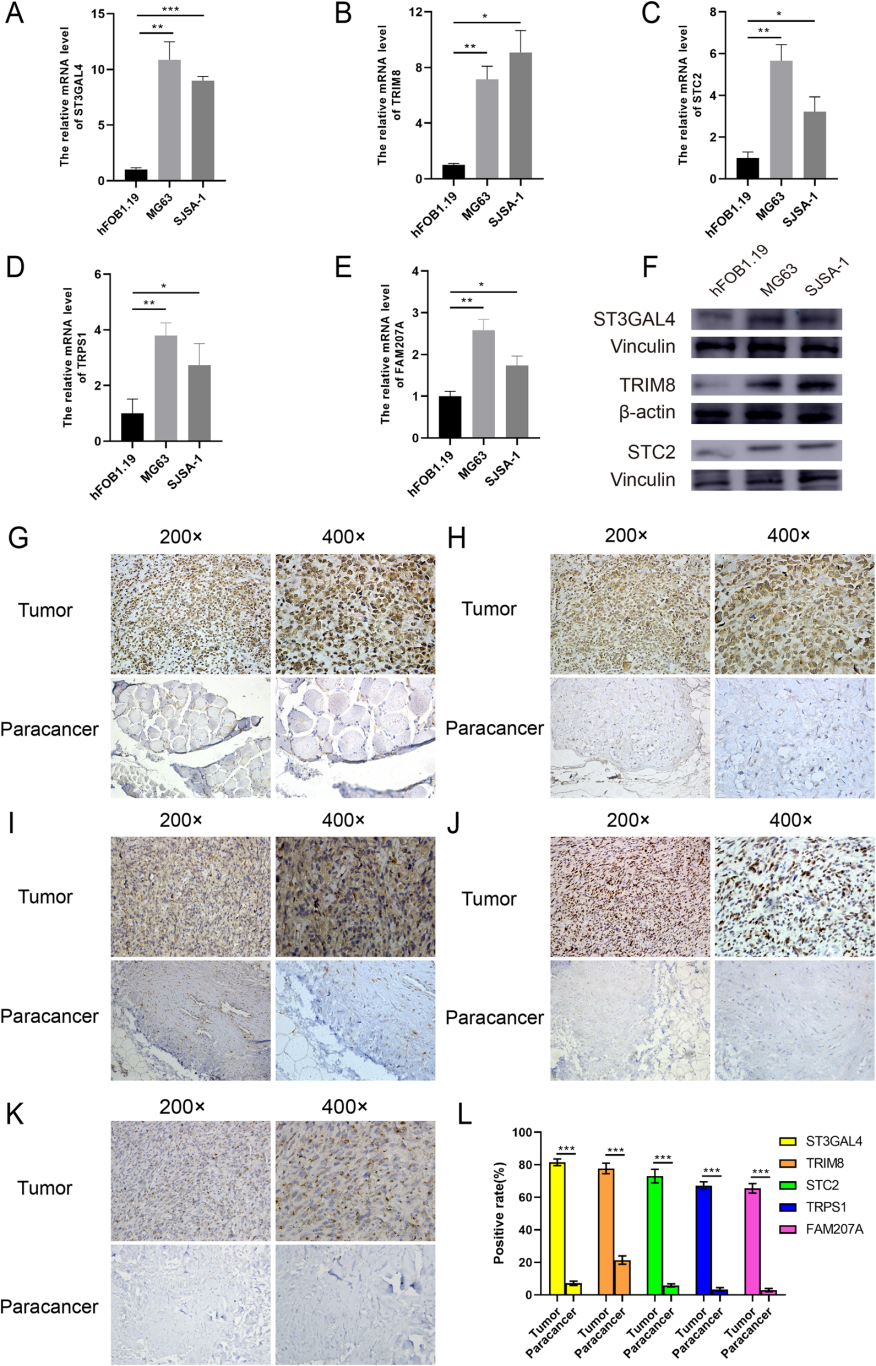
ST3GAL4, TRIM8, STC2, TRPS1, and FAM207A genes promote tumorigenesis in cells and patients. (**A**–**E**) Quantitative real-time PCR was performed to quantify the expression of ST3GAL4, TRIM8, STC2, TRPS1, and FAM207A genes in osteosarcoma cell lines (MG63 and SJSA-1) and a normal osteoblast cell line. (**F**) Western blot was performed to quantify the expression of ST3GAL4, TRIM8 and STC2 genes in osteosarcoma cell lines (MG63 and SJSA-1) and a normal osteoblast cell line. (**G**–**K**) The relative expression of ST3GAL4, TRIM8, STC2, TRPS1, and FAM207A genes, respectively, in the osteosarcoma tissue and the paracancerous tissues of the patient assessed by IHC. (**L**) The positive rates of ST3GAL4, TRIM8, STC2, TRPS1, and FAM207A genes in IHC pathology sections are quantified. (" * " represents P < 0.05, " ** " represents P < 0.01, and " *** " represents P < 0.001).

We performed qRT-PCR to detect the expression of ST3GAL4, TRIM8, STC2, TRPS1, and FAM207A genes in normal human osteoblasts (hFOB1.19) and osteosarcoma cells (MG63 and SJSA-1) (Fig. 7A–E). The results showed that the expression levels of ST3GAL4, TRIM8, STC2, TRPS1, and FAM207A genes were significantly higher in the osteosarcoma cell line compared to that in the normal human osteoblasts.

### Western blot analysis

We quantified the protein blots by the ImageJ software and found that the expression of ST3GAL4, TRIM8, and STC2 genes in both MG63 cells and SJSA-1 cells was higher than that in the hFOB1.19 cells, which further demonstrated that the expression of these three genes in osteosarcoma cells was higher than in osteoblasts (Fig. 7F). Uncropped blots are available in supplementary (Fig. S1).

### Immunohistochemistry

We observed all the immunohistochemically stained tissue sections under an inverted microscope, and the immunohistochemical staining results showed that the osteosarcoma tissue section and the paracancerous tissue section from the same patient were quite different, where the protein expression of the ST3GAL4, TRIM8, STC2, TRPS1, and FAM207A antibodies was found to be significantly higher in osteosarcoma compared to the expression of the antibodies in paracancerous tissues (Fig. 7G–K). Among them, the ST3GAL4, STC2, and FAM207A antibodies are mainly located in the cytoplasm, the TRIM8 antibody is mainly located in the nucleus and cytoplasm, and the TRPS1 antibody is mainly located in the cell nucleus. Then, we used the Image J software to analyze the positive rate of protein expression in the immunohistochemically stained images of these five genes, and through paired sample t-test in IBM SPSS Statistics software, we found that the positive rate of protein expression of these five genes in osteosarcoma tissues was significantly higher than that in paracancerous tissues (Fig. 7L, P < 0.05).

## Discussion

Osteosarcoma is a major public health concern because of its high incidence and a high degree of malignancy. Previous studies have demonstrated that the expansion of tumor cells depends on nutrient supply, and oxygen limitation is the primary factor controlling the formation of new blood vessels, glucose metabolism, survival, and the spread of tumors. Under hypoxic conditions, osteosarcoma cells can activate signal pathways that induce cell proliferation and angiogenesis to promote tumor growth rather than apoptosis. Additionally, hypoxia can induce osteosarcoma cells to transform into more aggressive types and increase their chemoresistance and metastatic ability^29,30^.

In this study, the expression profiles and survival data for OS were obtained using the UCSC Xena database (https://xena.ucsc.edu/) and the GTEx database (https://www.gtexportal.org/home). We downloaded the normal expression profile data. Additionally, we downloaded the GSE21257 dataset in the GEO database (https://www.ncbi.nlm.nih.gov/geo/) as a validation set for the prognostic model of osteosarcoma. We performed univariate Cox regression analysis, Lasso regression analysis, and multivariate Cox regression analysis to construct the osteosarcoma prognosis prediction model. We also performed principal component analysis and Kaplan–Meier survival analysis. A plot of the principal component analysis (Fig. 2E) based on the high-risk and low-risk groups showed that there was a separation between the patients in the high-risk and low-risk groups. The survival analysis showed a shorter survival interval for patients in the high-risk group, and similar results were obtained from the survival analysis in the validation dataset GES21257 (Fig. 2L), which indicated that the prognostic model was highly accurate. Besides, the results of the ROC diagnostic curve analysis, risk prediction analysis, column line plot, and calibration plot further confirmed the predictive accuracy of the model constructed in this study for osteosarcoma. Next, we used the CIBERSORT software to analyze the immune gene composition of the gene expression profile data. We found that the ST3GAL4, TRIM8, STC2, TRPS1, and FAM207A genes that we used to build the model were closely related to certain immune cells.

In this study, we conducted GO and KEGG analyses using hypoxia-related genes. We found that the GO entries were primarily distributed in the mitotic nuclear division, nuclear division, and extracellular matrix organization. Hypoxia is a vital physiological state promoting cancer invasion and progression. Besides, hypoxia can reduce anti-tumor immune activity and immunotherapy response^31^. Tumor cells adapt to hypoxia and simultaneously enhance the aggressiveness and promote the treatment-resistant phenotype of the tumor. Hypoxia can alter gene expression and eventually alter proteomics. These changes have several vital impacts on different cell activities, and hence, limit patient survival^32^. Tumor necrosis is considered to be an endpoint of serious chronic hypoxia. Due to the induction of hypoxia-related biomarkers, necrosis may enhance oxidative stress while altering the metabolic response. Additionally, hypoxia may also result in necrosis of the center of the tumor; in contrast, hypoxia causes apoptosis and suppresses necrosis during cancer development^33^. Moreover, the KEGG analysis revealed that the pathways of the hypoxia-related genes were primarily related to the cell cycle, the Rap1 signal transduction pathway, and the Hedgehog signal transduction pathway. Hypoxia has become a central problem in cancer treatment due to its multiple effects in cancer cell genome instability, tumor angiogenesis, invasiveness, metastasis, resistance to cell death, and metabolism. Interestingly, the Hedgehog signaling pathway plays an important role in enhancing bladder cancer progression and aggressiveness^34^. A group studied the occurrence, proliferation, and development of colorectal cancer through the Hedgehog signaling pathway^35^. The above finding was consistent with our results. We found that in addition to the hypoxia-related genes ST3GAL4, TRIM8, STC2, TRPS1, and FAM207A, the prognostic model constructed based on these five genes through multiple database analyses and screening showed that mortality was significantly higher in high-risk patients than in low-risk patients. Furthermore, the division between low-risk or high-risk groups led to different PCA clusters of the two groups. Figure 2E shows that the high-risk patients were separated from the low-risk patients. This further validated the model prediction accuracy for osteosarcoma in this study.

ST3GAL4 encodes for β-galactosidase α-2,3-sialyltransferase 4, which is involved in the biosynthesis of tumor antigens and is closely related to the occurrence and progression of cancer^36^. There are few studies on the relationship between ST3GAL4 and osteosarcoma hypoxia and osteosarcoma immunity. Interestingly, ST3GAL4 was negatively correlated with plasma cells and activated CD4^+^T memory cells. Previous reports have shown that these immune cells have an important inhibitory effect on tumor growth. Among these, plasma cells are an active regulator of immune response and protect against malignant tumors^37^. Activated CD4^+^T memory cells play a key role in generating effective anti-tumor immunity through multiple mechanisms such as enhanced antigen presentation, co-stimulation, T cell homing, T cell activation, and effector functions^38^. This finding is consistent with our research. Moreover, studies have shown that M0-enriched clusters decrease the recurrence-free survival and worsen the prognostic immune score^39^. Interestingly, our study found that ST3GAL4 is positively correlated with M0 macrophages. The M0 macrophages are closely related to the poor prognosis of melanoma, breast cancer, prostate cancer, and lung adenocarcinoma^39–42^.

TRIM8 is a commonly expressed factor in the Trim series, which, besides governing innate immune response, regulates numerous biological processes, such as cell survival, differentiation, and apoptosis^43^. According to previous reports, Trim8 regulates a wide range of biological processes, including development, differentiation, immune response, and cancer^44,45^. However, literature on the role of Trim8 in osteosarcoma and hypoxia is poor. Our study found that TRIM8 was negatively correlated with resting mast cells. Similar studies have found that innate and adaptive immune response cells, such as resting mast cells, have extensive connections with the external environment, can release inflammatory mediators and other factors that affect tumor growth, and inhibit cancer progression^28,41^. These observations are consistent with those found in our study.

STC2 codes for a glycoprotein associated with human cancer, which can enhance the aggressiveness of cancer. The expression of STC2 is strongly correlated with the development of human cancers and is a prognostic marker of renal, breast, and ovarian cancers^46^. Current evidence suggests that the activation of STC2 gene expression usually occurs in hypoxia, which is a common feature in the tumor microenvironment^47^. The change in the STC2 expression plays a potential role in the carcinogenesis of breast cancer and ovarian cancer^48^. A study predicted an association of STC2 with childhood osteosarcoma but with limited evidence. Here, we conducted a more detailed study and validated the expression of STC2 in osteosarcoma^49^. According to reports, CD8^+^T cells can directly kill tumor cells, including osteosarcoma ^50^. Additionally, CD8^+^ T cells also show a positive prognostic value in stage I–III colon cancer tumors^51^. We found that STC2 and CD8^+^ T cells showed a significant negative correlation trend, indicating that STC2 promoted the occurrence and development of osteosarcoma by inhibiting CD8^+^ T cells.

TRPS1 is one of the most significant hits in breast cancer cell lines^52^. Previous studies have shown the function of TRPS1 in tumorigenesis, such as colon cancer^53^, gastric cancer^54^, and breast cancer^55^, and TRPS1 is particularly tightly associated with breast cancer formation and progression. However, there is no relevant report on hypoxia in osteosarcoma. Our results showed that TRPS1 was negatively correlated with monocytes in osteosarcoma (R = –0.24). Previous studies have shown that monocytes effectively kill tumor cells through antibody-dependent and antibody-independent mechanisms and also prevent the metastasis and spread of cancer cells^56,57^. These studies also support our findings.

Only a few studies have been conducted on the protein-coding gene of FAM207A. For the first time, we conducted research on FAM207A related to hypoxia and tumor immunity. We found that FAM207A interacts with CD4^+^ memory T cells and ST3GAL4, and their activation was negatively correlated with *FAM207A* expression (*R* = –0.28), indicating that FAM207A is a potential tumor biomolecular target.

Previous reports have shown that the cytokines VEGF, IL-17, IL-6, IL-8, IL-1Ra, TNF-α, IL-34, and TGF-β play a role in the development of osteosarcoma. Among these, VEGF, IL-17, IL-6, IL-8, IL-1Ra, TNF-α, and IL-34 can promote cell proliferation in osteosarcoma; only TGF-β has a dual role in the tumor. In the early stage of cancer, TGF-β has an inhibitory effect on tumor growth, whereas it exerts a growth-promoting and invasion-promoting effect in the late stage of cancer^18,58^. We found that ST3GAL4, TRIM8, STC2, and FAM207A genes were positively correlated with VEGF, IL-6, IL-8, IL-1Ra, IL-34, and TGF-β cytokines. Therefore, we suggested that ST3GAL4, TRIM8, STC2, and FAM207A genes could regulate the immune microenvironment of osteosarcoma by influencing cytokines to promote tumor growth and invasion. TRPS1 was negatively correlated with TGF-β, possibly because TRPS1 suppressed the inhibitory effect of TGF-β during pre-tumor growth. Additionally, we performed a drug sensitivity analysis on these five genes and found that they have closely related sensitivity to numerous drugs. These findings provide a new reference for drug treatment of osteosarcoma.

Our findings suggested that ST3GAL4, TRIM8, STC2, TRPS1, and FAM207A could be used as potential biomarkers for the prognosis of patients with osteosarcoma.

Our research had certain shortcomings. First, the sample size was insufficient; we used only 396 normal samples and 88 osteosarcoma samples. Second, we did not conduct sufficient experiments to confirm our conclusions. Third, the mechanism of hypoxia and immune cell-related genes regulating osteosarcoma needs further investigation. In the future, basic experiments have to be performed with larger groups to further explore the underlying mechanisms of hypoxia and the effect of the tumor immune cell-related genes.

## Conclusion

We studied the effects of hypoxia and immune-infiltrating cells in osteosarcoma and provided new insights. ST3GAL4, TRIM8, STC2, TRPS1, and FAM207A are high-risk genes that are involved in the occurrence and development of osteosarcoma. These risk markers may be used as biomarkers to predict the prognosis of patients with osteosarcoma in clinical practice.

## Supplementary Information

Supplementary Information.

## Abbreviations

DEGs: Differentially expressed genes
GO: Gene ontology
KEGG: Kyoto encyclopedia of genes and genomes
OS: Overall survival
PCA: Principal component analysis
