# Supplementary material for: Five hypoxia and immunity related genes as potential biomarkers for the prognosis of osteosarcoma
Source: Sci Rep. 2022 Jan 31;12:1617. doi: 10.1038/s41598-022-05103-3 (PMC8804019; doi:10.1038/s41598-022-05103-3)
Supplement: Supplementary file 1 — Supplementary Information. [file 41598_2022_5103_MOESM1_ESM.docx]

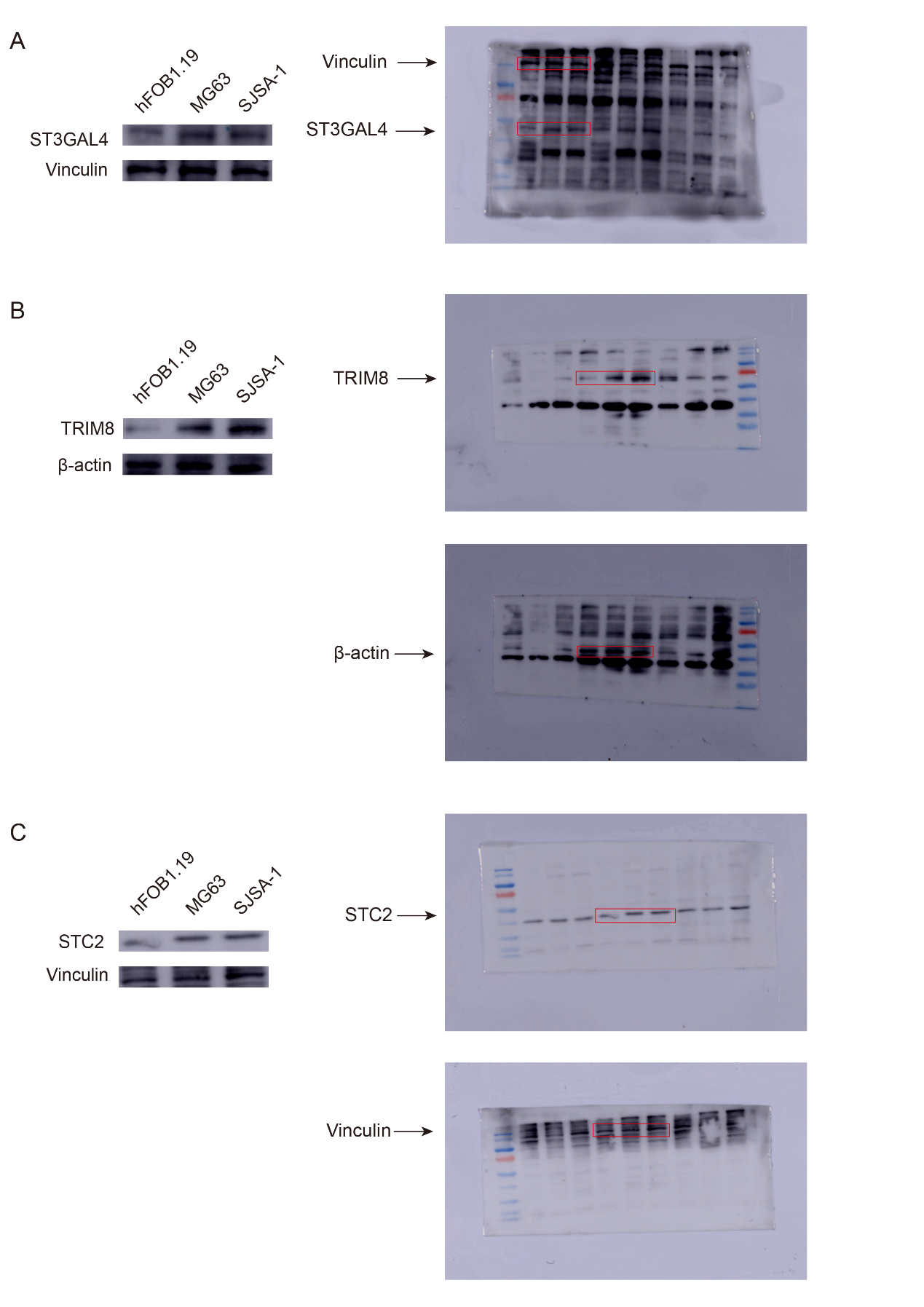


FIGURE S1 | Western blots shown in the main text (left panel), and original blots (right panel) of (A) ST3GAL4, (B) TRIM8 and (C) STC2.
